# A negative covariation between toxoplasmosis and CoVID-19 with alternative interpretations

**DOI:** 10.1038/s41598-020-69351-x

**Published:** 2020-07-27

**Authors:** Łukasz Jankowiak, Lajos Rozsa, Piotr Tryjanowski, Anders Pape Møller

**Affiliations:** 1 Institute of Biology, University of Szczecin, Wąska 13, 71-415 Szczecin, Poland; 2 GINOP Evolutionary Systems Research Group, Institute of Evolution, Centre for Ecological Research, Klebelsberg 3, Budapest, 8237 Hungary; 3 Institute of Zoology, Poznan University of Life Sciences, Wojska Polskiego 71C, Poznan, 60-625 Poland; 4 Ministry of Education Key Laboratory for Biodiversity Science and Ecological Engineering, College of Life Sciences, Beijing Normal University, Beijing, 100875 China; 5 Ecologie Systématique Evolution, Université Paris-Sud, CNRS, AgroParisTech, Université Paris-Saclay, 91405 Orsay Cedex, France

**Keywords:** Parasitology, Pathogens, Microbial ecology

## Abstract

Coronaviruses may exert severely negative effects on the mortality and morbidity of birds and mammals including humans and domestic animals. Most recently CoVID-19 has killed about half million people (27th of June, 2020). Susceptibility to this disease appears to differ markedly across different societies but the factors underlying this variability are not known. Given that prevalence of toxoplasmosis in human societies may serve as a proxy for hygiene, and it also exerts both direct and immune-mediated antiviral effects, we hypothesize a negative covariation between toxoplasmosis and measures of the CoVID-19 pandemic across countries. We obtained aged-adjusted toxoplasmosis prevalence of pregnant women from the literature. Since the differences in the CoVID-19 morbidity and mortality may depend on the different timing of the epidemics in each country, we applied the date of first documented CoVID-19 in each country as a proxy of susceptibility, with a statistical control for population size effects. Using these two indices, we show a highly significant negative co-variation between the two pandemics across 86 countries. Then, considering that the wealth of nations often co-varies with the prevalence of diseases, we introduced GDP per capita into our model. The prevalence of toxoplasmosis co-varies negatively, while the date of first CoVID-19 co-varies positively with GDP per capita across countries. Further, to control for the strong spatial autocorrelation among countries, we carried out a Spatial Structure Analyses of the relationships between the date of first CoVID-19, prevalence of toxoplasmosis, and GDP per capita. Results of this analysis did not confirm a direct causal relationship between toxoplasmosis and susceptibility to the CoVID-19 pandemics. As far as an analysis of observational data let us to suggest, it appears that the interaction between CoVID-19 and toxoplasmosis is mediated by GDP per capita and spatial effects. This prompts the question whether the formerly known covariations of CoVID-19 and BCG vaccination or air pollution might have also emerged as spurious indirect effects.

## Introduction

Coronaviruses are positive-stranded RNA viruses that may exert severely negative effects on the mortality and morbidity of a broad range of birds and mammals including humans and domestic animals. The strain called SARS-CoV-2 host-switched from bats to humans in Wuhan, China in November 2019 and subsequently gave rise to a devastating global pandemic called CoVID-19^1–3^. Susceptibility of human societies appear to be markedly heterogeneous ranging from modest to very high morbidity. Contrary to general expectations, more developed, wealthier communities living under better hygienic conditions appear to be more threatened than others. Thus, Austria is seemingly more severely hit than Hungary, the Czech Republic than Slovakia, and Israel than Palestine or Jordan.

Evidently, the first step to search for factors influencing this pandemic is to identify environmental correlates of different populations’ susceptibility. Sala and Miyakawa^4^ suggested that the different BCG vaccination policies across countries may partly explain differences in susceptibility to CoVID-19. Indeed, higher morbidity and mortality is observed in societies with no obligatory BCG vaccination. However, vaccination schemes tend to be uniform within countries, thus this hypothesis cannot explain the huge within-country differences that are often observed, such as those between Northern vs. Southern Italy. Zhu et al.^5^ described a covariation between exposure to air pollution and CoVID-19 infection.

We hypothesize that certain common infections coming together with a less hygienic lifestyle may trigger the human immune system and thus facilitate some protection against CoVID-19, an argument similar to the so-called ‘hygiene hypothesis’^6^. Toxoplasmosis is a candidate infection for this purpose because of two reasons. First, it is one of the most widespread latent infections of humanity^7,8^. As it does not transmit from human to human, its prevalence can be interpreted as a generalized index of group hygiene. Second, its causative agent, the eukaryotic protozoan *Toxoplasma gondii*, is known to exhibit at least some antiviral effects^9^.

*Toxoplasma gondii* is an intracellular parasite that infects birds and mammals as intermediate hosts, while the sexual phase of its life cycle can only be completed in feline definitive hosts, most often in domestic cats. It is distributed in human societies mostly by semi-domestic, partly-feral cats that depredate on infected rodents and birds and then eat their prey. Subsequently, the infective spores are released through their faeces and may get into direct contact with humans to cause infections. Alternatively, domestic animals may be infected by these spores and the consumption of their infected meat transmits *T. gondii* to humans. Thus, humans act like intermediate hosts, although they are not depredated by cats, and thus this is a dead-end for the parasites. ‘Luxury cats’ living on canned pet-food throughout their life may not transmit this infection. Asymptomatic infections are common in humans, especially among those living in the proximity of semi-feral domestic cats^10^.

*Toxoplasma gondii* excretes Dense Granule Protein-7 (GRA-7) into the host cell that inhibits viral replication. Its effect has been proven both in vitro and in vivo against indiana vesiculovirus, influenza A virus, Coxsackie virus, and herpes simplex virus. Overall, GRA-7 exhibits immune-stimulatory and a broad spectrum of antiviral activities via type I interferons signaling^9^. Moreover, in response to *T. gondii* infection, laboratory mice highly upregulate Immune Responsive Gene 1 in their lungs^11^. This is an interferon-stimulated gene that mediates antiviral effects against RNA viruses like the West Nile and Zika viruses through its product named itaconate^12^. It has been established that GRA-7 could be serve as alternative to treat tuberculosis^13^.

We need to emphasize, however, that the antiviral activities of *Toxoplasma gondii* are limited to the first, short and virulent phase of the infection, and not known to operate through the subsequent latent period that may last through the whole life of the host. Therefore, even in societies where a large proportion of the population carries latent toxoplasmosis, the proportion of infections actually expressing antiviral activities is very low. Thus we only claim that *Toxoplasma gondii* expresses at least some antiviral adaptations. Moreover, the apicoplast proteins of *Toxoplasma* are known to have immunogenic potential^14^.

Finally, we chose toxoplasmosis out of the candidate human infections partly because the availability of prevalence data from as many countries as possible. Unfortunately, as in the case of all other human infections, the methodologies of gathering and evaluating epidemiological data can be quite heterogeneous across countries. Below we set out to test whether there is a negative co-variation between levels of toxoplasmosis and CoVID-19 pandemic at a global scale.

## Results

**Figure 1:**
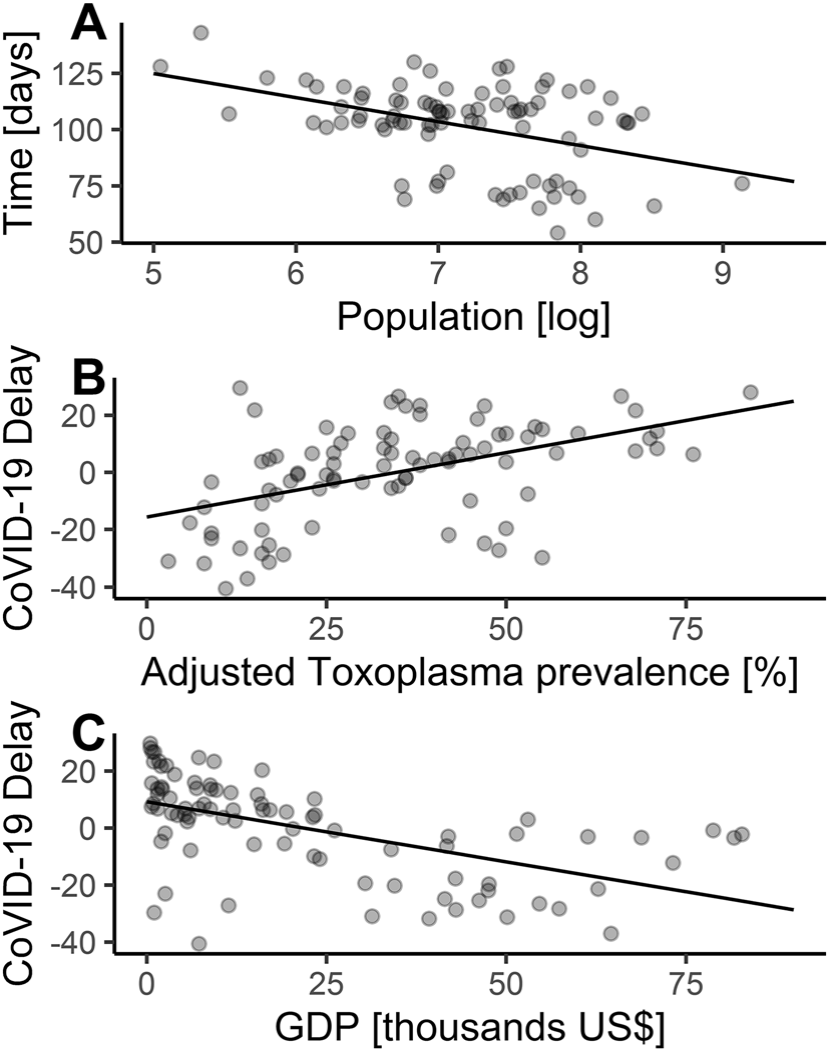
Linear regressions. (**A**) Starting date of epidemic counted since first case in China^26^ with relationship to population size of each country^25^. Residuals are used in (**B**) and (**C**) as dependent variable (CoVID-19 Delay). (**B**) CoVID-19 Delay in days (negative values = CoVID-19 faster, positive values = CoVID-19 later) and *Toxoplasma* prevalence. (**C**) CoVID-19 Delay and Gross Domestic Product per capita (GDP).

**Figure 2:**
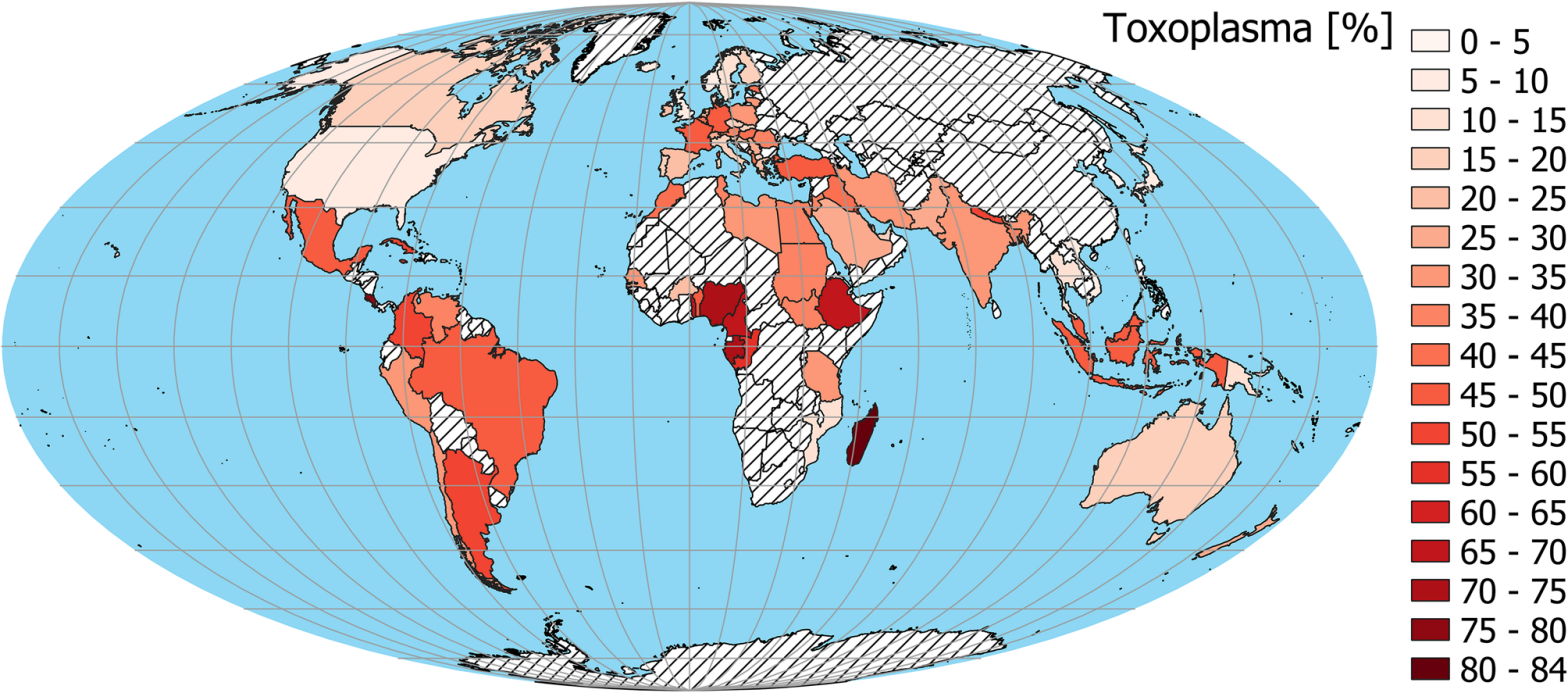
Spatial distribution of adjusted *Toxoplasma* prevalence^23^. China was not included in analysis because it was treated as 1st day case. The map was generated in QGIS software version 3.8.3-Zanzibar (https://www.QGIS.org)^33^.

**Figure 3:**
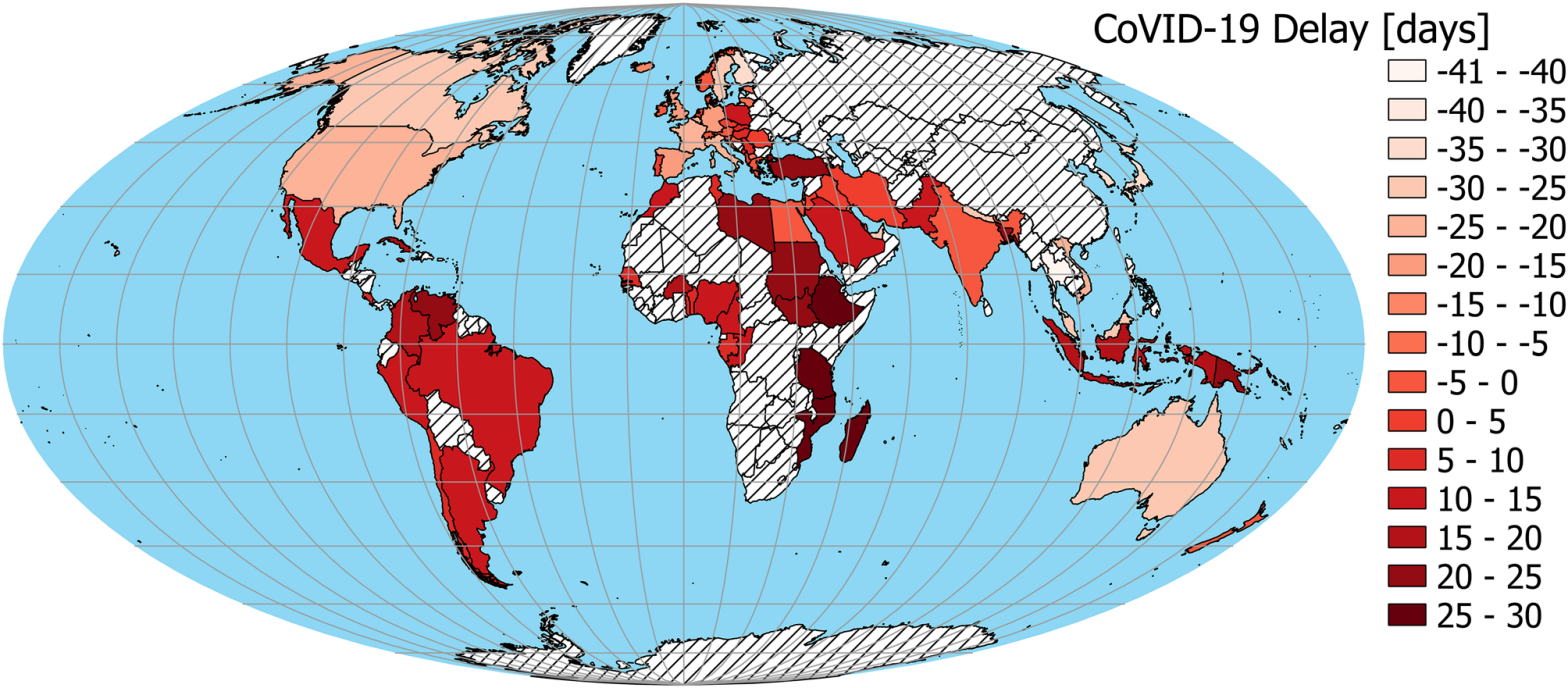
Spatial distribution of CoVID-19 Delay (negative values = CoVID-19 faster, positive values = CoVID-19 later). China was not included in the analysis because it was treated as 1^st^ day case. The map was generated in QGIS software version 3.8.3-Zanzibar (https://www.QGIS.org)^33^.

**Figure 4:**
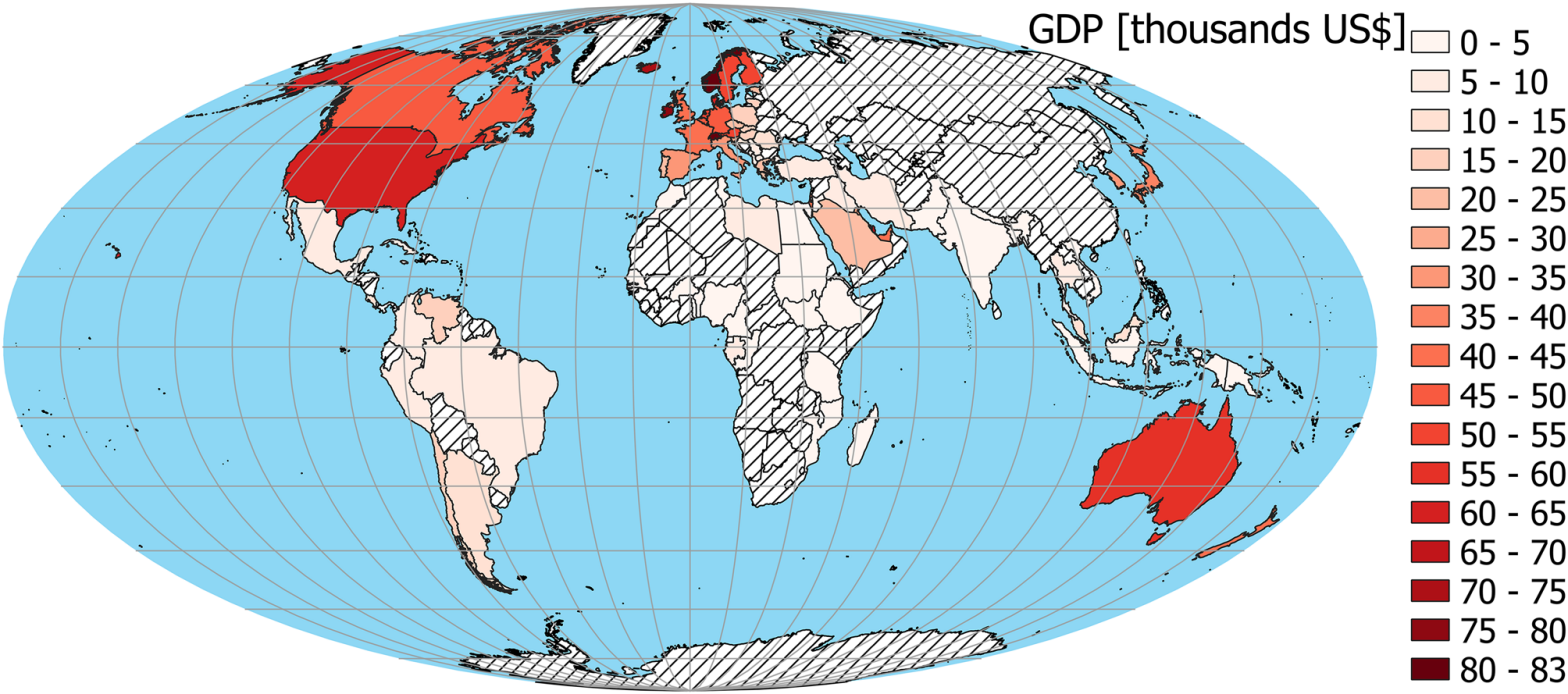
Spatial distribution of Gross Domestic Product (GDP)^28^ per capita. China not included in the analysis because it was treated as 1st day case. The map was generated in QGIS software version 3.8.3-Zanzibar (https://www.QGIS.org)^33^.

The linear regression model without a spatial component (Table 1, model 1, Fig. 1B,C) indicates that toxoplasmosis (N = 86; Fig. 2) is positively related to CoVID-19 Delay (Fig. 3), while GDP per capita (Fig. 4) is negatively related to CoVID-19 Delay. The total variation explained by these environmental variables is 36.8%.

**Table 1:** Linear regression models explaining CoVID-19 delay in different countries and due to toxoplasmosis and Gross Domestic Product (GDP) per capita.

	Estimate	Std. error	t value	P
**Model 1—without spatial covariate, AIC**_**c**_** = 701.163, r**^**2**^** = 0.368**				
Intercept	− 2.054	4.548	− 0.452	0.653
Toxoplasmosis	0.259	0.093	2.775	0.007
GDP	− 0.316	0.076	− 4.166	< .001
**Model 2—with spatial covarites**^a^**, AIC**_**c**_** = 682.523, r**^**2**^** = 0.523**				
Intercept	2.785	4.101	0.679	0.499
Toxoplasmosis	0.104	0.089	1.162	0.249
GDP	− 0.292	0.067	− 4.354	< .001

^a^For spatial covariates details, see Supplementary Information 1.

Results of the linear model with spatial covariates are presented in Table 1, model 2. Akaike information criterion indicates a better fit of this model. Total variation of all environmental variables plus the spatial variable is 52.3%. Variance partitioning indicates that the total variation explained by GDP per capita is 31.0%, by toxoplasmosis prevalence is 23.6%, and by the spatial component it is 39.5%. However, variation explained only by the spatial competent was 18.9%, only by GDP per capita was 10.8% (with the spatial component it was 13.2%) and by toxoplasmosis only it was 0.08% (with spatial component 5.9%).

## Discussion

As we predicted, there was a negative correlation between the prevalence of toxoplasmosis and the start CoVID-19 pandemics across countries that would be considered ‘highly significant’ by conventional measures. Moreover, as it is the case with many other diseases^15^, GDP per capita values co-vary negatively with toxoplasmosis prevalence. Opposite to this pattern, however, CoVID-19 emerged earlier in wealthier societies, most probably due to their more intensive participation of the global tourism and traffic industries.

Introducing a spatial component into the analysis, however, modified this result. It appears that CoVID-19 delay is greatly influenced by GDP per capita and spatial position of each country, while the effect of toxoplasmosis—contrary to our expectation—is weaker. This indicates that the strong negative covariation we have documented between prevalence of toxoplasmosis and the emergence of CoVID-19 on a global scale is likely to be a spurious side-effect mediated by GDP per capita and spatial effects. Perhaps the pathogen species, *Toxoplasma gondii*, we have chosen to investigate, or our methodologies, or the types of rough data we have analyzed were insufficient to verify it, or this interaction does not exist at all.

On the other hand, the above results does not falsify the hypothesis that CoVID-19 interacts negatively with other pathogens. The prevalence of toxoplasmosis may act as a proxy measure of group hygiene and our results may indicate a negative interaction of CoVID-19 with other, yet unidentified pathogens that are more widespread in resource-poor countries^16^.

Coronaviruses surround us, and we most probably get into unnoticed and symptomless contacts with human coronaviruses like HCoV-OC43 and HCoV-229E, and porcine and feline coronaviruses (PEDV, FCoV) quite frequently. We do not exclude the scenario that the variability in human populations’ resistance vs. susceptibility to the current CoVID-19 pandemic is influenced by such widespread but asymptomatic pathogens. Accordingly, a recent study indicated cross-reactivity with circulating common coronaviruses causing common cold^17^. Further, after the decline of the current CoVID-19 pandemic, more reliable indicators of population resistance vs. susceptibility will be available for our purposes, like the morbidity or mortality rates.

Our results may also serve as a cautionary note regarding the apparent interaction of other latent variables and the CoVID-19 pandemics. To date researchers found covariations of this pandemic with schemes of BCG vaccination^4,18^, vitamin D^19^, and levels of air pollution^20^. Although statistically highly significant, most of such studies were not controlled for GDP per capita effects, neither for geographical context^4^. Thus we cannot exclude the possibility that the apparent influence of BCG vaccination and air pollution on the current coronavirus pandemic is a spurious indirect effect, just as in our case with toxoplasmosis.

Different pathogen species utilizing the same host population as a shared resource tend to form an interactive pathogen community. Within this community, the ecological network of immune-mediated pathogen-pathogen interactions may define the emergence of disease^21^. How SARS-CoV-2 will be positioned in the global ecological network of human pathogens (probably the most species-rich pathogen community on Earth^22^) is yet to be seen.

## Methods

Toxoplasmosis is routinely screened in pregnant women in several countries, thus nation-level prevalence values are available from the literature. We obtained data from Flegr and Dama^23^ (Fig. 2). Since the prevalence of this parasite is known to be age-dependent, these values were adjusted to a standard age following Lafferty^24^.

Currently, the CoVID-19 pandemic is still in its growing phase in many countries, but possibly saturated or already declining in others. Thus the actual measures of morbidity or mortality would be misleading to compare among countries. Therefore, we compared the starting date, i.e. the date of the first documented case of the CoVID-19 disease in each country. For obvious reasons, countries with large population sizes are more likely to have earlier dates of the first case of the disease. To control for population size differences, we applied residuals taken from the (first date ~ log [population size]) regression (Fig. 1A; β_0_ = 178.241 ± 18.258 s.e, β_log(population)_ = − 10.673 ± 2.527 s.e., t value = − 4.223, P < 0.001). Positive residuals mean that countries had later start of the pandemic as expected (more resistant), while negative residuals indicate that countries had earlier starting dates (more susceptible). This variable is interpreted as a population-size-corrected time delay of first documented case of CoVID-19 in each country, and hereafter we called it ‘CoVID-19 Delay’ (Fig. 3). Population sizes of each country were taken from UNData^25^, with values of countries that recently split treated accordingly. The first date of disease from CoVID-19 in each country originated from WHO^26^.

The prevalence of numerous human diseases depends on GDP per capita as an estimate of resource availability, and hence the ability to live a healthy life without exposure to zoonoses, and/or in the absence of untreated diseases^27^. To control for this, both in the case of toxoplasmosis and CoVID-19, we introduced Gross Domestic Product (GDP 2018) per capita (Fig. 4), derived from World Bank data^28^.

Further, statistical autocorrelation is a general feature of ecological variables measured across geographic space^29^. Since this effect, called spatial autocorrelation, violates the assumption of the independence of data required by most standard statistical procedures, we applied a Spatial Structure Analyses of the relationships between the date of first CoVID-19 documented case (controlled for human population size), prevalence of toxoplasmosis (age adjusted), and GDP per capita.

Using a linear regression model, we analyzed the relationship between these three variables. We found no issue with multicollinearity between toxoplasmosis prevalence and GDP per capita (VIF = 1.325). However, there was negative spatial correlation (Pearson’s r = − 0.495, Corrected Pearson’s r F = 14.598, Corrected D.F. = 44.921, p < 0.001; Figs. 2 and 4) between these two variables.

We tested all used variables for spatial autocorrelation with Moran’s local indicator (Moran’s I)^29^. Indices > 0 indicate that pairs of locations are more similar, those < 0 indicate pairs that are less similar than expected by random pairs of observations. Moran’s Is were computed for 11 distance classes and were showed on spatial correlograms. Analyzes of these correlograms indicate (Supplementary Information 1) significant spatial similarity of neighboring countries according to toxoplasmosis prevalence, CoVID-19 Delay and GDP per capita (Figs. 2, 3, 4). In order to account for spatial autocorrelation, we used spatial eigenvector mapping (SEVM)^30^. This tool allows us to select an eigenvector (spatial filters) that minimalizes Moran’s I in model residuals. Then selected filters can be used as explanatory variables in the linear model. We used partial regression analyses to quantify how much of the variation of the response variable is explained by the spatial structure versus by the environmental variables.

All analyses were computed using SAM software^30^. Regression graphs were plotted using ggplot2 package^31^ in R statistical software^32^. Maps were created using QGIS software (version 3.8.3-Zanzibar)^33^.

## Supplementary information

Supplementary information.
