# Supplementary material for: A negative covariation between toxoplasmosis and CoVID-19 with alternative interpretations
Source: Sci Rep. 2020 Jul 27;10:12512. doi: 10.1038/s41598-020-69351-x (PMC7385593; doi:10.1038/s41598-020-69351-x)
Supplement: Supplementary file 1 — Supplementary information. [file 41598_2020_69351_MOESM1_ESM.docx]

**Title:**

Strong negative covariation between toxoplasmosis and CoVID-19 at a global scale: a spurious indirect effect?

**Author names and affiliations**:

**Łukasz Jankowiak^1^, Lajos Rozsa^2^, Piotr Tryjanowski^3^ and Anders Pape Møller^4,5^**

^1^ Institute of Biology, University of Szczecin, Wąska 13, 71-412, Szczecin, Poland; e-mail: jankowiakl@gmail.com

^2^ GINOP Evolutionary Systems Research Group, Institute of Evolution, Centre for Ecological Research, Budapest, Hungary; e-mail: lajos.rozsa@gmail.com

^3^ Institute of Zoology, Poznan University of Life Sciences, Poznan, Poland, e-mail: piotr.tryjanowski@gmail.com

^4^ Ministry of Education Key Laboratory for Biodiversity Science and Ecological Engineering, College of Life Sciences, Beijing Normal University, Beijing 100875; China; e-mail: anders.moller@u-psud.fr

^5^ Ecologie Systématique Evolution, Université Paris-Sud, CNRS, AgroParisTech, Université Paris-Saclay, F-91405 Orsay Cedex, France

**Corresponding author:** Łukasz Jankowiak <jankowiakl@gmail.com >

Table S1. Linear regression models explaining CoVID-19 delay in different countries

| Model 2 – with spatial covaraites, AIC_c_ = 682.523 | | | |  |
| --- | --- | --- | --- | --- |
|  | Estimate | Std. Error | t value | P |
| Intercept | 2.785 | 4.101 | 0.679 | 0.499 |
| Toxoplasmosis | 0.104 | 0.089 | 1.162 | 0.249 |
| GDP | -0.292 | 0.067 | -4.354 | <.001 |
| Spatial Filter 3 | 50.529 | 13.934 | 3.626 | <.001 |
| Spatial Filter 4 | -39.319 | 12.254 | -3.209 | 0.002 |
| Spatial Filter 6 | 22.704 | 11.953 | 1.899 | 0.061 |
| Spatial Filter 10 | -20.593 | 12.028 | -1.712 | 0.091 |
| Spatial Filter 16 | -23.548 | 11.952 | -1.97 | 0.052 |

Fig S1. Spatial structure analyse - correlogram of CoVID-19 delay (which are residuals and should be interpreted as population size corrected time delay of first case of COVID-19 in given country)

Fig S2. Spatial structure analyse - correlogram of Gross Domestic Product (GDP) per capita.

Fig S3. Spatial structure analyse - correlogram of variable Toxoplasmosis prevalence.
